# Toward the Discovery of a Novel Class of Leads for High Altitude Disorders by Virtual Screening and Molecular Dynamics Approaches Targeting Carbonic Anhydrase

**DOI:** 10.3390/ijms23095054

**Published:** 2022-05-02

**Authors:** Amena Ali, Abuzer Ali, Musarrat Husain Warsi, Mohammad Akhlaquer Rahman, Mohamed Jawed Ahsan, Faizul Azam

**Affiliations:** 1 High Altitude Research Center, Taif University, P.O. Box 11099, Taif 21944, Saudi Arabia; 2 Department of Pharmaceutical Chemistry, College of Pharmacy, Taif University, P.O. Box 11099, Taif 21944, Saudi Arabia; 3 Department of Pharmacognosy, College of Pharmacy, Taif University, P.O. Box 11099, Taif 21944, Saudi Arabia; abuali@tu.edu.sa; 4 Department of Pharmaceutics and Industrial Pharmacy, College of Pharmacy, Taif University, P.O. Box 11099, Taif 21944, Saudi Arabia; mvarsi@tu.edu.sa (M.H.W.); mrahman@tu.edu.sa (M.A.R.); 5 Department of Pharmaceutical Chemistry, Maharishi Arvind College of Pharmacy, Jaipur, Rajasthan 302 039, India; jawedpharma@gmail.com; 6 Department of Pharmaceutical Chemistry & Pharmacognosy, Unaizah College of Pharmacy, Qassim University, Unaizah 51911, Saudi Arabia; f.azam@qu.edu.sa

**Keywords:** high-altitude disorders, carbonic anhydrase, virtual screening, molecular dynamics, DFT

## Abstract

For decades, carbonic anhydrase (CA) inhibitors, most notably the acetazolamide-bearing 1,3,4-thiadiazole moiety, have been exploited at high altitudes to alleviate acute mountain sickness, a syndrome of symptomatic sensitivity to the altitude characterized by nausea, lethargy, headache, anorexia, and inadequate sleep. Therefore, inhibition of CA may be a promising therapeutic strategy for high-altitude disorders. In this study, co-crystallized inhibitors with 1,3,4-thiadiazole, 1,3-benzothiazole, and 1,2,5-oxadiazole scaffolds were employed for pharmacophore-based virtual screening of the ZINC database, followed by molecular docking and molecular dynamics simulation studies against CA to find possible ligands that may emerge as promising inhibitors. Compared to the co-crystal ligands of PDB-1YDB, 6BCC, and 6IC2, ZINC12336992, ZINC24751284, and ZINC58324738 had the highest docking scores of −9.0, −9.0, and −8.9 kcal/mol, respectively. A molecular dynamics (MD) simulation analysis of 100 ns was conducted to verify the interactions of the top-scoring molecules with CA. The system’s backbone revealed minor fluctuations, indicating that the CA–ligand complex was stable during the simulation period. Simulated trajectories were used for the MM-GBSA analysis, showing free binding energies of −16.00 ± 0.19, −21.04 ± 0.17, and −19.70 ± 0.18 kcal/mol, respectively. In addition, study of the frontier molecular orbitals of these compounds by DFT-based optimization at the level of B3LYP and the 6-311G(d,p) basis set showed negative values of the HOMO and LUMO, indicating that the ligands are energetically stable, which is essential for forming a stable ligand–protein complex. These molecules may prove to be a promising therapy for high-altitude disorders, necessitating further investigations.

## 1. Introduction

High-altitude pulmonary edema (HAPE), high-altitude cerebral edema (HACE), and acute mountain sickness (AMS) are all manifestations of high-altitude sickness, which occurs at elevations as a result of hypoxia. Unlike AMS, which is self-limiting, HAPE and HACE are real crises that require immediate clinical intervention and management [1]. The primary cause of high-altitude sickness is a reduction in oxygen supply at high elevations, which leads to hypoxemia. 

Carbonic anhydrase (CA) inhibition has been found to be an effective treatment strategy for high-altitude sickness [2]. According to the report of West [3], CAs inhibition causes diuresis, and bicarbonate excretion results in moderate metabolic acidosis. In addition, both arterial oxygenation and ventilatory control can be augmented by CA inhibition. Breathing is also improved by inhibiting CA in peripheral chemoreceptors by decreasing hypoxic and hypercapnic sensitivity [4]. Thus, acute mountain sickness symptoms may be alleviated by reducing pulmonary vasoconstriction and altering the cerebral blood flow [5]. In experimental animals, the intraventricular injection of acetazolamide, one of the most effective CAIs, has been demonstrated to curb the increase in bicarbonate concentration in the cerebrospinal fluid and, hence, alter the pH of the CSF during respiratory acidosis [6]. Interestingly, Parati and colleagues reported that, after 6 h and 2 days of advent at high altitudes, the systemic resting diastolic and mean arterial blood pressures were elevated in comparison to the sea level. Acetazolamide therapy prevented an increased blood pressure, which might be a result of decreased sympathetic activation and increased NO generation [7]. In line with this, low-dose acetazolamide pretreatment on the day of ascent to a high altitude was also shown to lower systemic blood pressure [8]. Acetazolamide has also been shown to have a positive effect on periodic breathing during sleeping at high altitudes. Acetazolamide significantly reduced apnea-associated hypoxemia and enhanced the sleep quality, which may help to reduce periodic breathing while sleeping [4]. Therefore, acetazolamide represents the most widely used and well-studied medication for treating high-altitude conditions [9]. Hence, potential carbonic anhydrase-targeting agents should be developed, owing to acetazolamide’s therapeutic value in high-altitude-related diseases.

Drug development has long been recognized as a sophisticated, costly, time-consuming, and challenging endeavor. On average, it is estimated that a new medication discovery requires around 12 years and 2.7 billion USD via the typical drug development pipeline [10]. Lowering the cost of research and accelerating the development process of new drugs has become a pressing issue for the pharmaceutical sector. Computer-aided drug discovery has evolved as a formidable and intriguing technique for designing drugs more quickly, cheaply, and effectively. Since the successful development of the HIV protease inhibitor nelfinavir in the USA in 1997, computational techniques have become a vital tool in drug discovery initiatives and a cornerstone for new drug development methodologies [11,12]. Recently, the fast rise of computational methods for drug discovery such as pharmacophore modeling, virtual screening, molecular dynamics, and quantum chemical computations has had a substantial and unprecedented influence on drug designs and yielded valuable insights into various therapeutic regimens [13,14,15].

In this study, we used three pharmacophore models based on diverse scaffolds comprising 1,3,4-thiadiazole, 1,3-benzothiazole, and 1,2,5-oxadiazole and screened them against the ZINC database. Precise drug–enzyme interactions were studied by molecular docking. Three potential hits in the complex with CA were further subjected to an all-atom molecular dynamics simulation in order to understand thermodynamic stability in physiological environments. MM/GBSA computations were performed on snapshots of simulated trajectories to calculate the binding energies. DFT computations were performed to optimize the geometry and analyze the HOMO/LUMO energies. Finally, ADME parameters were also predicted to find their suitability as drug candidates.

## 2. Result and Discussion

**Figure 1:**
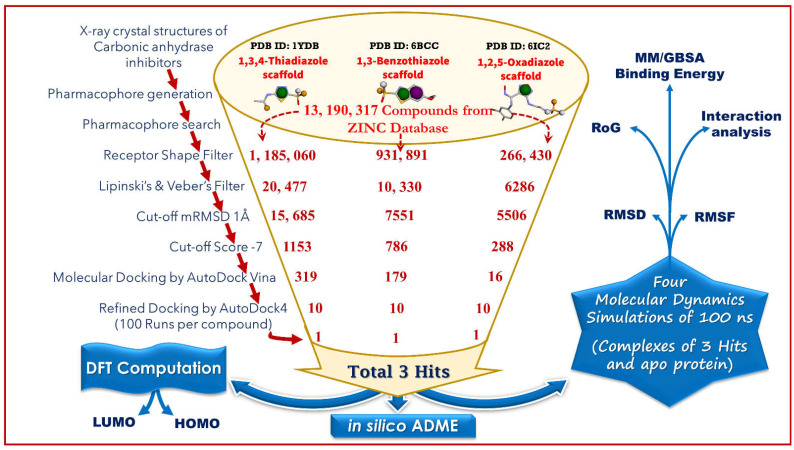
An illustration of the computational approaches implemented in this study.

As illustrated in Figure 1, a systematic computational method was adopted, which involved pharmacophore modeling, virtual screening, density functional theory-based calculations, molecular docking, and molecular dynamics simulation.

### 2.1. Generation of Pharmacophore Model and Virtual Screening

**Figure 2:**
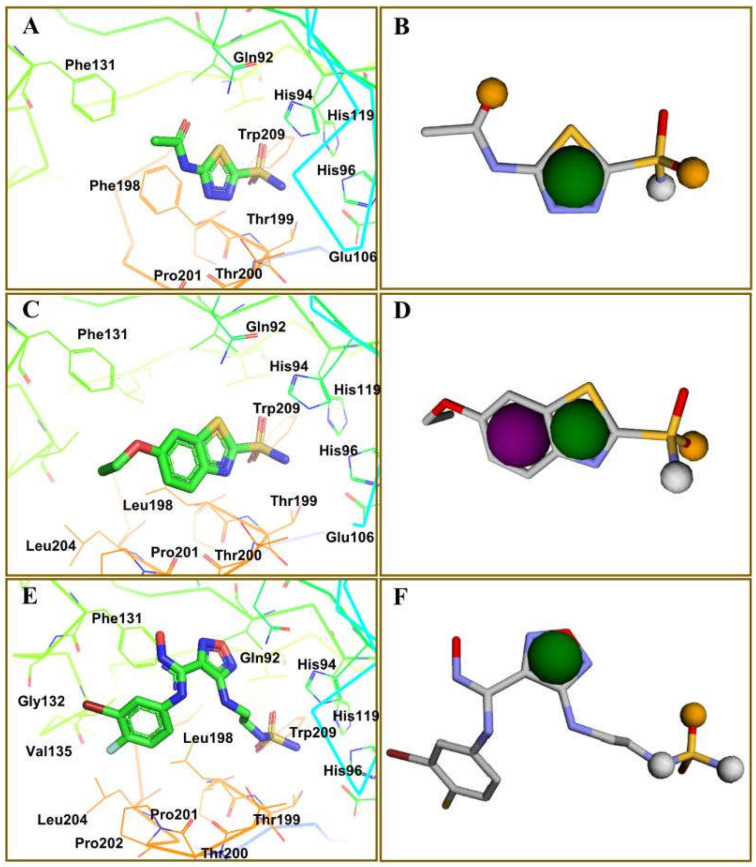
Key interacting residues involved in the intermolecular interactions of co-crystallized inhibitors of carbonic anhydrase with the 1,3,4-thiadiazole (PDB: 1YDB (**A**)), 1,3-benzothiazole (PDB: 6BCC (**C**)), and 1,2,5-oxadiazole (PDB: 6IC2 (**E**)) scaffolds. Common pharmacophoric features are presented (**B**,**D**,**F**).

Among several isoforms of CA, acetazolamide exerts a maximum inhibitory effect against CA-II (a comparative affinity against several CA isoforms is listed as Table S1 in the Supplementary Information). Therefore, CA-II bound to different inhibitors were included in this study. A pharmacophore is a representation of the spatial and electronic properties required for interaction with a macromolecular target, which results in a pharmacological response. Three ligand-based pharmacophore models based on the crystal structure of CA-II co-crystallized with acetazolamide (5-acetamido-1,3,4-thiadiazole-2-sulfonamide), ethoxzolamide (6-ethoxy-1,3-benzothiazole-2-sulfonamide), and epacadostat (N-(3-bromo-4-fluorophenyl)-N′-hydroxy-4-{[2-(sulfamoylamino)ethyl]amino}-1,2,5-oxadiazole-3-carboximidamide) bearing PDB IDs 1YDB, 6BCC, and 6IC2, respectively, were developed in this study using the Pharmit server [16], which offers a setup for the virtual screening of databases utilizing suitable pharmacophore features. Only four key properties of ligands in terms of hydrogen bond acceptors, hydrogen bond donors, hydrophobicity, and aromaticity were chosen to construct an effective pharmacophore query for virtual screening (Figure 2).

The ZINC database [17], containing 13,190,317 compounds, was screened using each generated pharmacophore model as a filter to afford 1,185,060, 931,891 and 266,430 hits from the 1,3,4-thiadiazole, 1,3-benzothiazole, and 1,2,5-oxadiazole scaffolds, respectively. A shape query was enabled in the next step, which applies the receptor shape constraints to the pharmacophore-aligned pose, eliminating compounds that match the pharmacophore but have significant steric clashes with the receptor, yielding 20,477, 10,330, and 6286 hits. Lipinski’s and Veber’s filters were used to further refine the number of hits, resulting in 15,686, 7551, and 5506 molecules from each model. Lipinski’s parameters were implemented as follows: a molecule with a molecular mass less than 500 Da, no more than ten hydrogen bond acceptors, no more than five hydrogen bond donors, and a logP value of less than five for the octanol–water partition coefficient. However, Veber’s filter considered ≤10 rotatable bonds and a ≤140 Å total polar surface area.

In the next step of screening, the AutoDock Vina scoring function and Smina, a clone of AutoDock Vina with improved minimization capability, were enabled in the Pharmit server for energy minimization to optimize both the position and conformation of the generated hits with regard to the input receptor. Minimized molecules acquire a conformation at the local minimum obtained via a gradient descent on the energy surface, starting with the initial query-aligned poses. A cut-off minimized RMSD (mRMSD) of 1Å further reduced the hits to 1153, 786, and 288 molecules, whereas a cut-off score of −7 kcal/mol finally afforded 319, 179, and 16 compounds. The mRMSD determined how much the compound deviated from the original query, whereas compounds with unfavorable binding energies were eliminated by implementing strict −7 kcal/mol criteria.

### 2.2. Molecular Docking by AutoDock Vina

**Figure 3:**
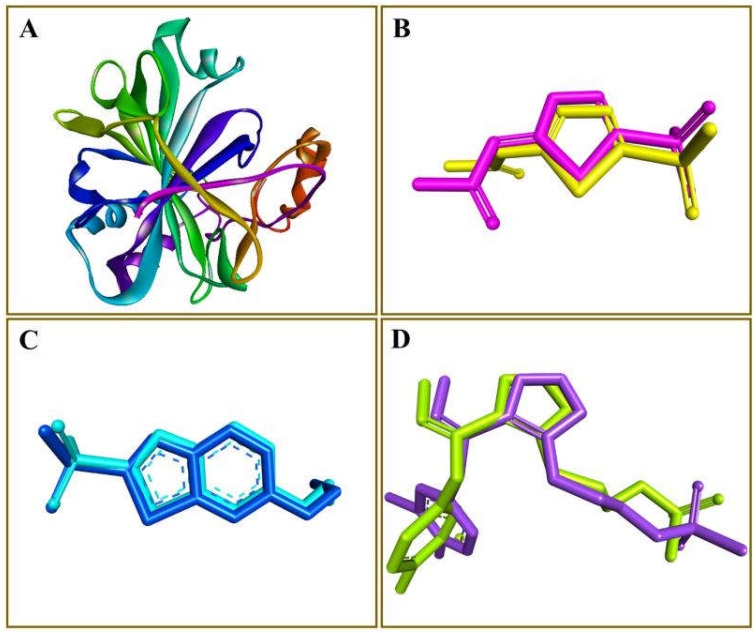
Validation of the docking protocol. The carbonic anhydrase protein is shown as a ribbon (**A**), whereas the co-crystallized ligands along with docked conformations of acetazolamide (**B**), ethoxzolamide (**C**), and epacadostat (**D**) are presented in a stick style.

The 319, 179, and 16 hits identified through acetazolamide, ethoxzolamide, and epacadostat-based pharmacophore models, respectively, were subsequently screened using AutoDock Vina for in-depth molecular docking studies. A python script was utilized to automate the docking of several ligands against CA in a single click. Prior to the screening of all ligands, co-crystallized inhibitors of PDB-1YDB, 6BCC, and 6IC2 were extracted and redocked back into their active site. The RMSD values were calculated between the crystallographic pose and the best-docked conformation. The RMSD values of acetazolamide, ethoxzolamide, and epacadostat were found to be 0.40, 0.33, and 0.26 Å, respectively (Figure 3). The smaller RMSD value implies that the docking methodology may be trustworthy for final docking investigations of the representative compounds against CA. The docking data demonstrated the likely binding modalities in the CA’s active site and yielded the highest docking scores. AutoDock 4.2 was used to refine the next-level docking of ten top-ranked compounds from each series.

### 2.3. Docking Refinement by AutoDock 4.2

Although AutoDock Vina’s binding mode predictions are faster and more accurate than AutoDock 4.2, noncovalent bonding analyses show that the amount of hydrogen bonds formed between a ligand and a receptor determined by the latter is better correlated with the experiment than the former. Moreover, the AutoDock 4.2 method yields a higher correlation coefficient with respected experiments than the Vina method. Therefore, the top ten compounds in each pharmacophore series identified by AutoDock Vina were designated for an exhaustive docking analysis by AutoDock 4.2. With a hundred independent runs, thirty compounds in total were docked against CA using the Lamarckian genetic algorithm approach for a rigid protein and a flexible ligand. The docking scores are presented in Tables S2–S4 of the Supplementary Information. Finally, the top three drug candidates were identified as ZINC12336992 or 4-[(2R)-2-(5,6-dimethyl-7-oxo-1H-pyrazolo [1,5-a]pyrimidin-2-yl)pyrrolidine-1-carbonyl]-3-fluorobenzamide, ZINC24751284 or 3-[3-(2,3-Dihydroindol-1-yl)-3-oxopropyl]-2-oxo-1,3-benzoxazole-6-sulfonamide, and ZINC58324738 or (2R)-N-carbamoyl-2-(5,7-dihydrobenzo[d][2]benzazepin-6-yl)propenamide, screened through pharmacophore models derived from the 1,3,4-thiadiazole, 1,3-benzothiazole, and 1,2,5-oxadiazole scaffolds, respectively (Table 1). Compared to the co-crystal ligands of PDB-1YDB, 6BCC, and 6IC2, these molecules had the highest docking scores of −9.0, −9.0, and −8.9 kcal/mol, respectively.

Acetazolamide, ethoxzolamide, and epacadostat, as co-crystal ligands of 1YDB, 6BCC, and 6IC2, respectively, demonstrate hydrogen bonding with common amino acid residues His94 and Thr199 in the active site of CA [18,19] (Figures S1–S3 of the Supplementary Information). Gln92 and His96 are also known to contribute hydrogen bonds in acetazolamide and ethoxzolamide, respectively. However, epacadostat reveals additional HBs with His96, His119, and Thr200 [20]. Other residues present in the binding site include His64, Val121, Phe131, Val143, Phe198, Pro201, Pro202, and Trp209. Zn(II) ion is present in the active site of CA and is known to interact with the inhibitor through His94, His96, and His119 residues [21].

**Figure 4:**
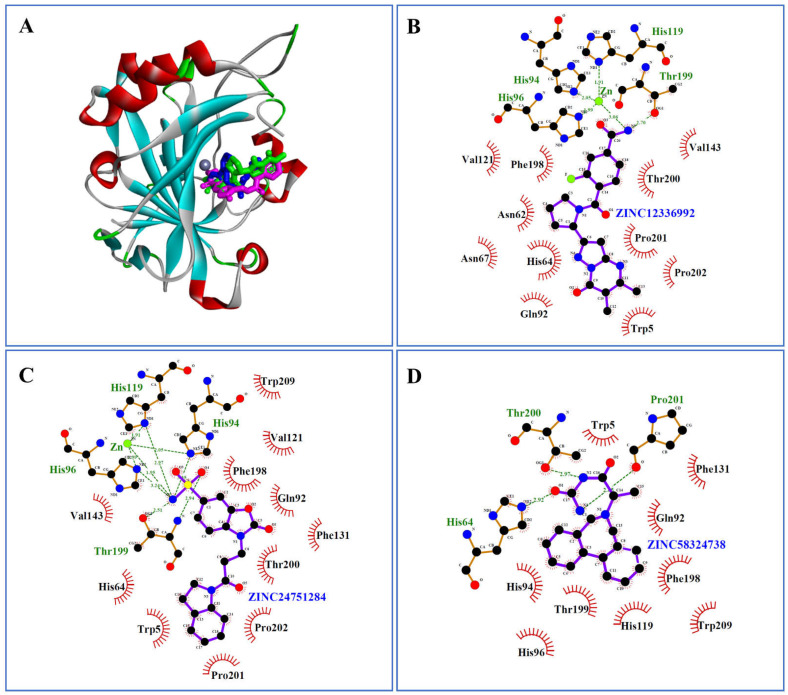
(**A**) Intermolecular complex formed between carbonic anhydrase-II (shown as a ribbon) and ZINC12336992, ZINC24751284, and ZINC58324738, shown as stick styles in green, magenta, and blue, respectively. Zn(II) is represented as a grey ball in the binding pocket. (**B**–**D**) Ligplot diagrams showing non-bond interactions of the top three hits.

Interestingly, ZINC12336992 and ZINC24751284 coordinated through Zn(II) ion via the His94, His96, and His119 residues (Figure 4B,C). In contrast, there was no connection between ZINC58324738 and the Zn(II) ion in the inhibitor binding cavity (Figure 4D). However, His64, Thr200, and Pro201 contributed hydrogen bonds with ZINC58324738. Apart from these, the docked compounds were supported by Trp5, Asn62, Val121, Phe131, Val143, Phe198, and Trp209 in terms of hydrophobic contacts.

### 2.4. Molecular Dynamics Simulation

#### 2.4.1. Analysis of the Root Mean Square Deviation

**Figure 5:**
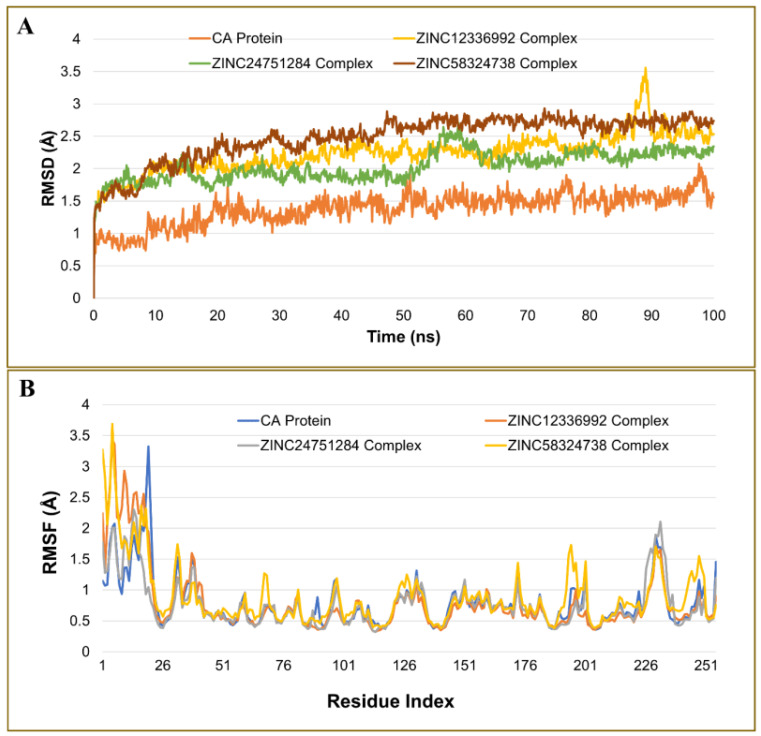
Plot of molecular dynamics simulation trajectories of carbonic anhydrase apo protein, and protein–ligand complexes during a 100-ns MD simulation showing the root mean square deviation (RMSD) (**A**) and root mean square fluctuation (RMSF) (**B**).

The stability and convergence of ZINC12336992, ZINC24751284, and ZINC58324738 in complex with CA were studied using a 100-ns molecular dynamics (MD) simulation. The root mean square deviation (RMSD) values of the backbone atoms were computed, as shown in Figure 5A. The findings implied that all complexes remained in equilibrium over the simulation period. The RMSD values of the apo protein and the ZINC12336992-, ZINC24751284-, and ZINC58324738-bound complexes ranged between 0.68 and 2.07, 1.25 and 3.56, 1.14 and 2.71, and 1.2 and 2.93 Å, respectively. The Cα-RMSD for the ZINC24751284-CA complex had a smaller fluctuation than the ZINC12336992 and ZINC58324738 systems, showing average RMSD of 2.04 ± 0.23, 2.24 ± 0.3, and 2.47 ± 0.35 (standard deviation) Å, respectively. Thus, when complexed with ZINC24751284, the CA structure is more stable. Furthermore, the secondary structure did not show a significant alteration in the proteins during the simulations, according to the protein–ligands and protein trajectory assessment.

#### 2.4.2. Analysis of Residue Mobility

The root mean square fluctuations (RMSF) of the Cα atoms of the protein were determined and illustrated in Figure 5B to evaluate the dynamics of the essential residues in the complexes compared to the uncomplexed form. In both the bound and unbound states, the terminal residues fluctuated the most. The most fluctuation in the apo protein was seen with Phe20 displaying 3.32 Å, which decreased upon binding with ZINC12336992, ZINC24751284, and ZINC58324738, showing 1.94, 1.05, and 1.62 Å, respectively. The average fluctuation of unbound CA was noted as 0.79 ± 0.4 (standard deviation) Å, which was declined upon binding with ZINC24751284, displaying 0.76 ± 0.38 Å. However, the ZINC12336992 and ZINC58324738 complexes demonstrated higher fluctuations than the apo protein, showing 0.78 ± 0.54 Å and 0.88 ± 0.5 Å. In general, the flexibility of the corresponding residues in the drug-bound complexes was somewhere closer to the native unbound CA. Furthermore, the analysis of the RMSF revealed that the values of the vital residues involved in the intermolecular interactions such as Gln92, His94, His96, His119, Phe131, Val143, Phe198, Thr199, Thr200, Pro201, Pro202, and Trp209 were at the bottom of the curve. These low-fluctuating residues promoted the stabilization of the docked molecules at the binding site.

#### 2.4.3. Radius of Gyration Analysis

**Figure 6:**
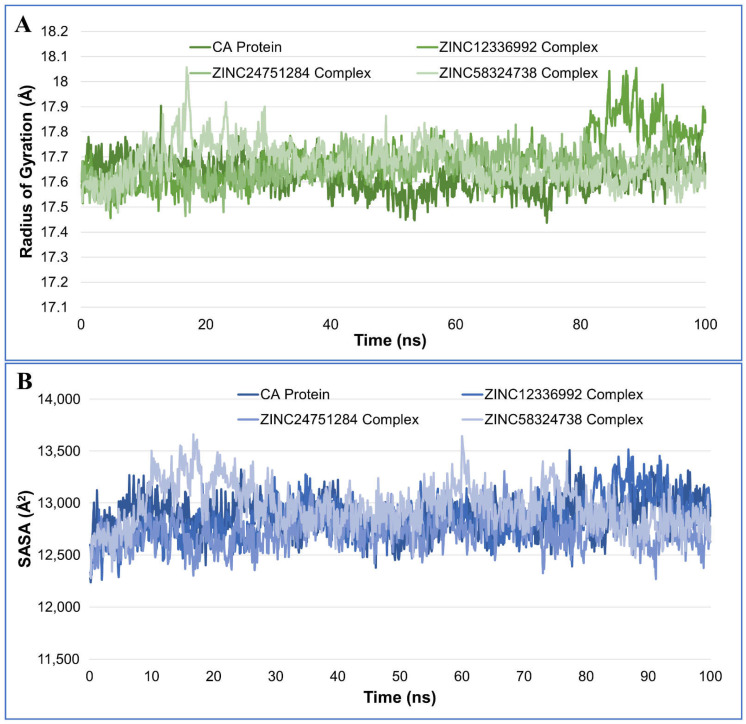
Plot of the radius of gyration (**A**) and solvent-accessible surface area (SASA) (**B**), during the 100-ns molecular dynamics simulation of the carbonic anhydrase protein and complex of ZINC12336992, ZINC24751284, and ZINC58324738.

The radius of gyration (RoG) is an indicator of the compactness and size of protein molecules. The RoG may be used to measure the folding and unfolding of protein structures when ligands are bound. The RoG values for the drug-bound complexes were generally closer to the native unbound CA (Figure 6A). The average RoG values for CA, CA-ZINC12336992, CA-ZINC24751284, and CA-ZINC58324738 were recorded as 17.62 ± 0.06, 17.68 ± 0.10, 17.66 ± 0.06, and 17.67 ± 0.08 Å, respectively. A Higher RoG indicates that the protein–ligand association is less compact or more unfolded. Nevertheless, if the protein’s RoG value remains constant during the MD simulation, it is said to be stably folded [22]. The value of RoG is regarded as unfolded if it varies with time. As demonstrated in Figure 6A, when compared to the unbound protein, each complex displayed very comparable behaviors in terms of the compactness and nearly constant values of RoG.

#### 2.4.4. Solvent-Accessible Surface Area Analysis

The solvent-accessible surface area (SASA) of the protein in the absence and presence of ligands was also investigated. The computation of SASA of the protein–ligand complex aids in predicting the extent of conformational changes that the aqueous solvent can access [23]. Therefore, the SASA was used to evaluate interactions between the complex and the solvent throughout the 100-ns MD simulation. The plot of the SASA vs. simulation time for the unbound protein and protein–ligand complexes is shown in the Figure 6B. The average SASA values for CA, CA-ZINC12336992, CA-ZINC24751284, and CA-ZINC58324738 were 1287.85 ± 17.74, 1284.71 ± 19.68, 1272.42 ± 15.89, and 1296.10 ± 21.95 nm^2^, respectively. Upon ZINC58324738 binding, the SASA rises slightly as a result of a portion of the bound ligand surface protruding outside of the protein surface, forming an extended surface. The binding of ZINC12336992 and ZINC24751284, on the other hand, resulted in reduced SASA values, because the surface becomes unexposed to the solvent after ligand binding.

#### 2.4.5. Hydrogen Bond Analysis

**Figure 7:**
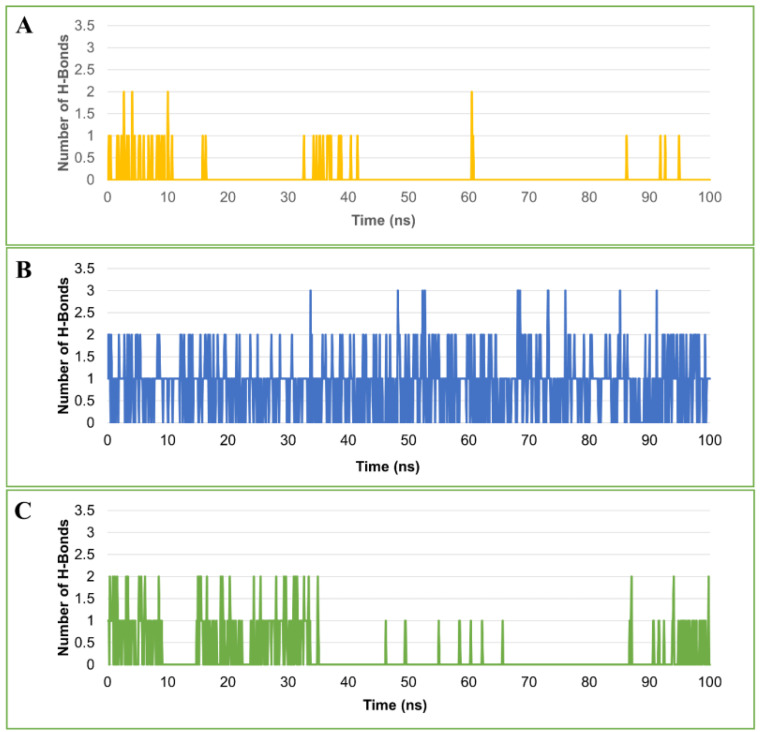
Plot of number of hydrogen bonds observed in the complex of carbonic anhydrase and ZINC12336992 (**A**), ZINC24751284 (**B**), and ZINC58324738 (**C**) during the 100-ns molecular dynamics simulation.

**Figure 8:**
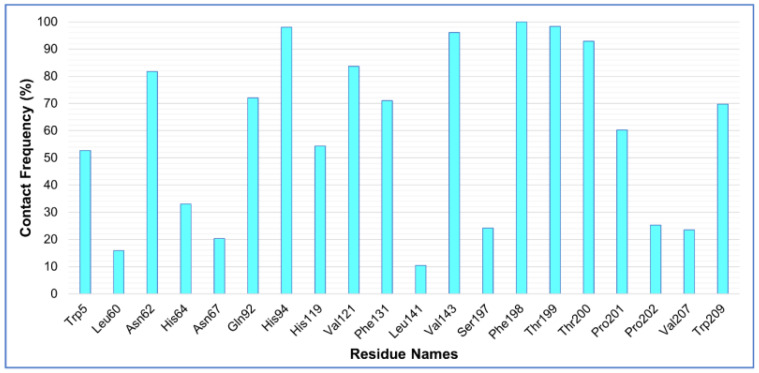
Hydrogen bond contact frequency of ZINC24751284 in a complex with carbonic anhydrase during the 100-ns molecular dynamics simulation.

The stability of the protein–ligand complex is facilitated by the formation of hydrogen bonds between the receptor and ligand. It also plays a role in drug design in terms of specificity, metabolization, and adsorption. Hence, hydrogen bonds formed by each ligand–protein complex were investigated. Figure 7 displays the total number of hydrogen bonds observed in the complexes after the 100-ns simulation time. In the CA-ZINC12336992 and CA-ZINC58324738 complexes, one to two hydrogen bonds were identified. CA-ZINC24751284, on the other hand, was shown to form two to three hydrogen bonds, two of which were consistently observed throughout the simulation period. Furthermore, as presented in Figure 7A,C, both compounds ZINC12336992 and ZINC58324738 displayed a nonuniform hydrogen-bonding pattern during the entire simulation period. Through the above-detailed H-bond analysis, we can conclude that the compound ZINC24751284 was bound to the CA more effectively and tightly when compared to the other two compounds. A favorable platform for polar interactions such as oxazolidinone, an amide functionality of the indoline ring, and a sulfonamide group is uniquely appended in ZINC24751284. Figure 8 displays the contact frequency of the hydrogen bonds in the CA-ZINC24751284 complex.

#### 2.4.6. Binding Energy Estimation by MM/GBSA Method

The binding free energy (ΔG) of the simulated complex was computed to confirm the inhibitor affinity of the CA–ligand complexes predicted by the docking investigations. The MD trajectories were used in the estimation of the binding free energies. The total of both the polar and nonpolar solvation and the sum of the electrostatic energies, van der Waals energies, and internal energies for bonded interactions were determined for all the complexes using the MM/GBSA method and are reported in Table 2. According to the MM/GBSA-predicted binding energy, ZINC24751284 had a maximum affinity with CA, followed by ZINC58324738 and ZINC12336992, showing −21.04 ± 0.17, −19.70 ± 0.18, and −16.00 ± 0.19 kcal/mol, respectively.

### 2.5. Density Functional Theory Computations

**Figure 9:**
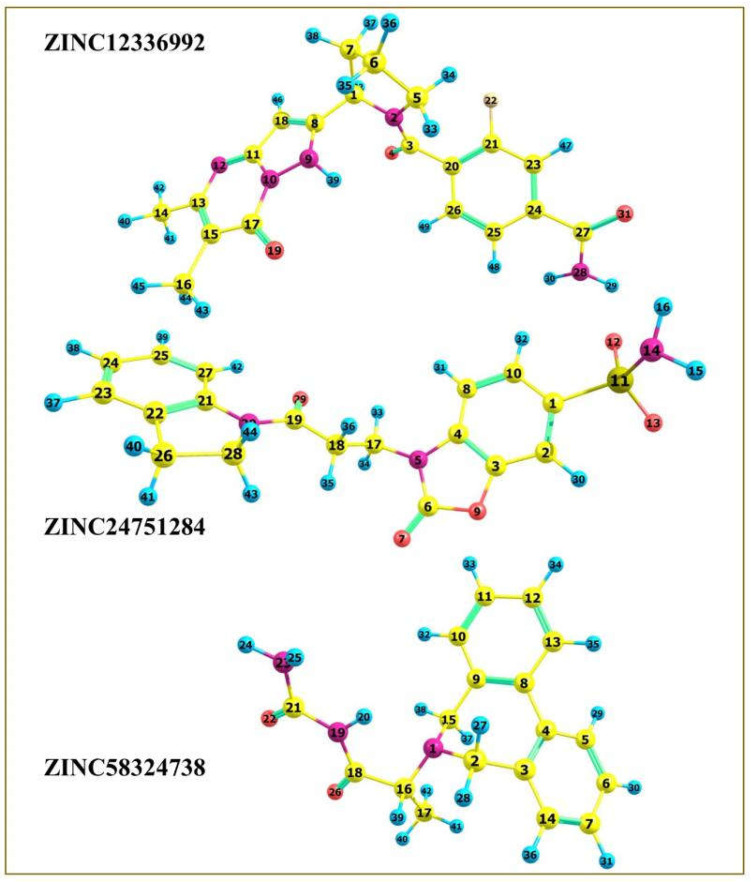
DFT-optimized geometry of the top three hits.

The drug’s frontier molecular orbitals explain the charge–transfer interactions with the protein binding site. Therefore, the top three hits were subjected to density functional theory (DFT) computations using the Orca 5.0.2 program [24,25] to comprehend the electronic and energetic states better. The geometry of compounds ZINC12336992, ZINC24751284, and ZINC58324738 was optimized at the level of the 6-311G(d,p) basis set using the Lee–Yang–Parr correlation functional (B3LYP) method (Figure 9). The Cartesian coordinates of the optimized compounds are shown in Tables S5, S7 and S9, and the bond properties are displayed in Tables S6, S8 and S10.

**Figure 10:**
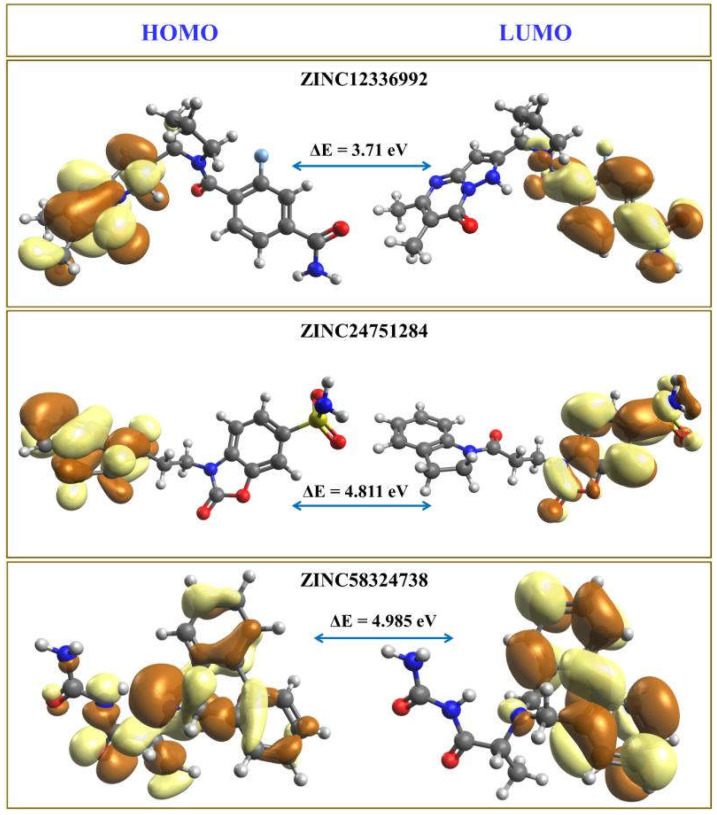
HOMO and LUMO orbitals of the top three hits. The positive and negative phases of the molecular orbitals are represented by the brown and light-yellow colors, respectively.

It is easy to extract an electron from the electron-rich HOMO, since the orbitals are at their maximum energy, while adding electrons to the lowest-lying orbital in the electron-deficient LUMO appears to be the most energy-efficient [26,27]. The electron-acceptor and -donor characteristics of the molecules under investigation are denoted by the HOMO and LUMO, respectively. The HOMO–LUMO energy gap is the difference in the HOMO and LUMO energy levels representing an electron’s excitation from the ground state HOMO to the first excited state LUMO [28]. The HOMO–LUMO energy gap is a numerical depiction of the reactivity and stability of the molecules. Figure 10 shows the computed HOMO and LUMO energies and the energy gap (ΔE) values. The brown and light-yellow colors depict the positive and negative phases of the molecular orbitals, respectively.

The compounds’ HOMO–LUMO orbitals are structured according to electron localization inside the molecule, and the energy is confined to specific orbitals. The amide-substituted benzene ring of ZINC12336992 is surrounded by LUMO, whereas HOMO mainly engulfs the pyrazolopyrimidine moiety. In ZINC24751284, the benzoxazole moiety is covered by the LUMO, and the HOMO is located on the dihydroindolyl ring. The HOMO represents azepine and parts of the carbamoyl moieties in ZINC58324738, and the LUMO is part of the benzene rings flanking the azepine fragment. In general, negative HOMO and LUMO values imply high stability and are necessary to form a stable ligand–protein association. The higher the influence on intermolecular charge transfer and bioactivity, the smaller the gap between HOMO and LUMO energies. As a result of the large energy gap, an electron’s ability to travel from the HOMO to LUMO is impeded, resulting in the inhibitor’s poor affinity for the target protein. The ΔE values of ZINC12336992, ZINC24751284, and ZINC58324738 were measured as 3.71 eV, 4.81 eV, and 4.99 eV, respectively, which are significantly lower values, indicating the potential bioactivity of the hit compounds. Interestingly, ZINC12336992, showing the maximum MM/GBSA-computed binding energy, had a minimum HOMO–LUMO gap. In contrast, the most potent hit, ZINC24751284, demonstrated a moderate gap, whereas the maximum HOMO–LUMO energy difference was noticed in the moderately active compound ZINC58324738.

### 2.6. ADME Analysis

The SwissADME program [29] was used to compute the ADME parameters. The compounds’ molecular weights ranged from 309.36 to 397.40 (≤500), with the log *p* values estimated to be between 1.62 and 2.52 (≤5.00). The H-bond acceptors were estimated to be between 3 and 6 (≤10) in number, whereas the H-bond donors were reported to be between 1 and 2 (≤5) in number. The total polar surface areas (TPSA) of ZINC12336992, ZINC24751284, and ZINC58324738 were calculated as 113.56, 133.99, and 75.43 Å^2^, and the percentage of absorption (%Abs) was expected to be 69.82%, 62.77%, and 82.98%, employing the equation %Abs = 109−0.345 × TPSA [30,31]. TPSA, or the surface of polar atoms, is a feature that has been demonstrated to correlate strongly with the passive transport of compounds via biomembranes, allowing an estimation of the drug transport characteristics in the gut, as well as blood–brain barrier passage. Lower molecular flexibility (determined by the number of rotatable bonds), modest polar surface area, or total hydrogen bond count (sum of donors and acceptors) are all indicators of better oral bioavailability [32,33]. It is widely accepted that around half of the experimental medications never reach the clinic due to inadequate pharmacokinetic properties. A successful oral medicine is rapidly and thoroughly absorbed from the gastrointestinal tract, delivered precisely to its site of action in the body, metabolized in a manner that does not entirely abolish its activity, and removed in a manner that is safe for the organs. The number of rotatable bonds was estimated to be between four and five. In order to pass the oral bioavailability standards, the number of rotatable bonds must be less than 10 [32]. The lipophilicity of all the compounds was determined to be adequate for GIT absorption. Fortunately, none of the compounds compromised the Lipinski rule of five [34], making them appropriate drug candidates against CA inhibition. Table 3 enlists the estimated ADME profile.

## 3. Materials and Methods

Figure 1 illustrates the methods used in this study.

### 3.1. Virtual Screening

The Pharmit server software [16] (http://pharmit.csb.pitt.edu; accessed on 20 December 2021) was used to generate the pharmacophore models based on the X-ray crystal structures of the 1,3,4-thiadiazole, 1,3-benzothiazole, and 1,2,5-oxadiazole scaffolds bound to carbonic anhydrase with PDB IDs 1YDB, 6BCC, and 6IC2, respectively (https://www.rcsb.org; accessed on 18 December 2021). Each pharmacophore was individually used for virtual screening against the ZINC database [17], comprising 13,190,317 purchasable compounds. A number of filtering criteria were applied, such as the pharmacophore shape filter, exclusive shape constraint with a tolerance of 0.5, Lipinski’s “rule of five” [34], and Veber’s [33] filter, which includes a molecular weight ≤ 500, rotatable bonds ≤ 10, logP ≤ 5, polar surface area ≤140 Å^2^, hydrogen bond acceptors ≤ 10, and hydrogen bond donors ≤ 5. Finally, an additional scoring function, namely the minimized RMSD (mRMSD) that is the RMSD between the query-aligned pose and the minimized pose, was also applied, and a cut-off mRMSD was set to 1 Å.

### 3.2. Molecular Docking by AutoDock Vina

The pharmacophore model based on the 1,3,4-thiadiazole, 1,3-benzothiazole, and 1,2,5-oxadiazole scaffolds afforded 319, 179, and 16 hits, respectively, which were docked into the inhibitor binding cavity of the carbonic anhydrase enzyme using the AutoDock Vina program [35]. A grid box of 25, 25, and 25 Å was placed at −5.308, 3.249, and 15.595 Å in the x, y, and z directions, respectively, with grid spacing of 1 Å. The rest of the parameters were kept at the program’s default. Open Babel was used for converting the ligand files from sdf format to pdbqt format [36]. A python-based script was used for the automated docking and scoring of the 514 total hits obtained in previous step.

### 3.3. Molecular Docking by AutoDock 4.2

The top 30 hits, consisting of 10 compounds obtained from each query pharmacophore, were further subjected to pose refinement by using AutoDock 4.2 [37]. Each compound was docked into the inhibitor-binding cavity of carbonic anhydrase in a 100-run protocol implementing Lamarckian genetic algorithm methodology.

### 3.4. Molecular Dynamics Simulation

The top hit from each query pharmacophore in complex with carbonic anhydrase obtained after molecular docking by AutoDock 4.2 was further subjected to the molecular dynamics simulation. The system was prepared using the web-based CHARMM-GUI [38,39,40] interface with the CHARMM36 force field [41]. All the simulations were performed using the NAMD 2.13 package [42]. The TIP3P explicit solvation model was used, and the periodic boundary conditions were set with dimensions of 10 Å, 10 Å, and 10 Å in the x, y, and z, directions, respectively. The parameters for the top docking results were generated using the CHARMM general force field. Afterward, the system was neutralized using the appropriate (Cl^−^/Na^+^) ions. The MD protocols involved minimization, equilibration, and production. A 2fs time step of integration was chosen for all MD simulations, and the equilibration was carried in the canonical (NVT) ensemble, while the isothermal–isobaric (NPT) ensemble was implemented for the production. Through 100 ns of the MD production, the pressure was set at 1 atm using the Nose’–Hoover Langevin piston barostat [43,44] with a Langevin piston decay of 0.05 ps and a period of 0.1 ps. The temperature was set at 298.15 K using the Langevin thermostat [45]. Short-range nonbonded contacts had a distance cut-off of 12 Å with a pair list distance of 16 Å, while the Lennard Jones interactions were smoothly terminated at 8 Å. The particle mesh Ewald (PME) approach [46,47] was employed to model long-range electrostatic interactions, with all simulation cells in a grid spacing of 1 Å. The SHAKE method was used to restrict all covalent connections containing hydrogen atoms. We used the same procedure for all MD simulations to ensure uniformity. In addition, a fourth simulation was also performed with carbonic anhydrase in an unbound state in order to understand the conformational changes observed upon ligand binding.

### 3.5. Binding Energy Calculations

The one-average molecular mechanics generalized born surface area (MM/GBSA) approach [48] implemented in the MOLAICAL code was used for the relative binding energy calculations [49], in which the ligand (*L*) binds to the protein receptor (*R*) to form the complex (*RL*):

$$\Delta G_{bind}=\Delta G_{RL}-\Delta G_{R}-\Delta G_{L}$$

which can be represented by contributions of different interactions:

$$\Delta G_{bind}=\Delta H-T\Delta S=\Delta E_{MM}+\Delta G_{Sol}-T\Delta S$$

where the changes in the gas phase molecular mechanics (Δ*E_MM_*), solvation Gibbs energy (Δ*G*_sol_), and conformational entropy (−*T*Δ*S*) are determined as follows: Δ*E_MM_* is the sum of the changes in the electrostatic energies Δ*E_ele_*, the van der Waals energies Δ*E_vdW_*, and the internal energies Δ*E_int_* (bonded interactions); Δ*G_Sol_* is the total of both the polar solvation (calculated using the generalized Born model) and the nonpolar solvation (calculated using the solvent-accessible surface area); and −*T*Δ*S* is calculated by the normal mode analysis. The solvent dielectric constant of 78.5 and the surface tension constant of 0.03012 kJ mol^−1^ Å^2^ were used for the MM/GBSA calculations.

### 3.6. Density Functional Theory Computations

The density functional theory (DFT) calculations were carried out using Orca 5.0.2 software [24,25]. The optimization was accomplished by opting the basis set 6-311G(d,p) utilizing the Lee–Yang–Parr correlation functional (B3LYP) [50,51]. The output file of the DFT-optimized structure was handled in Chemcraft, a graphical software for the visualization of quantum chemistry computations (https://www.chemcraftprog.com; accessed on 12 February 2022). Avogadro software was used to depict the highest occupied molecular orbital (HOMO) and the lowest unoccupied molecular orbital (LUMO) [52,53].

### 3.7. Pharmacokinetic Properties of the Top-Scoring Molecules

SWISS ADME software was used to predict the ADME profile of the title compounds [29].

## 4. Conclusions

In this investigation, diverse and robust computer-aided drug discovery strategies such as pharmacophore modeling, virtual screening, density functional theory-based computations, molecular docking, and molecular dynamics simulations were sequentially employed against carbonic anhydrase to select the most active compounds from the ZINC database for tackling high-altitude-related diseases. DFT-based optimization of the top hits revealed negative values for the HOMO and LUMO, indicating that the molecules are energetically stable, which is required to form a stable ligand–protein complex. The pharmacokinetic investigation indicated that the prospective compounds did not have any issues and could be used in further in vitro/in vivo studies. Finally, the results of the MM/GBSA binding energy estimations using simulated trajectories validated the docking results and the stability of the putative inhibitors in a complex with CA, warranting further experimental research. The top three hits, ZINC12336992, ZINC24751284, and ZINC58324738, were recognized as prospective CA-targeting molecules that might be developed as possible treatments for high-altitude disorders.

## Figures and Tables

**Table 1:** Top three hits obtained after virtual screening of the ZINC database.

SN	Pharmacophore	Top Hits	Chemical Structure	Docking Score (kcal/mol)
1.	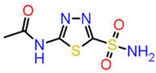	ZINC12336992	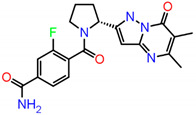	−9.0
2.	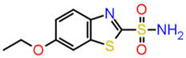	ZINC24751284	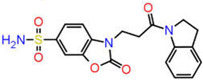	−9.0
3.	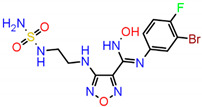	ZINC58324738	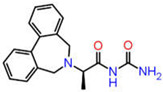	−8.9

**Table 2:** Calculated binding free energies of the top three hits (kcal/mol).

Complex	Δ*G*	Δ*E_(electrostat.)_* + Δ*E_(sol.)_*	Δ*E_(VDW)_*
ZINC12336992	−16.00 ± 0.19	8.3899	−24.3925
ZINC24751284	−21.04 ± 0.17	10.9063	−31.9482
ZINC58324738	−19.70 ± 0.18	9.5605	−29.2571

**Table 3:** ADME prediction of the top three hits.

Parameters	Compounds		
	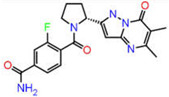 ZINC12336992	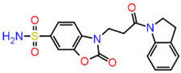 ZINC24751284	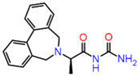 ZINC58324738
Molecular weight	397.40	387.41	309.36
No. H-bond acceptor	5	6	3
No. H-bond donor	2	1	2
Log P_O/W_ (iLOGP)	2.52	1.62	1.65
No. rotatable bonds	4	5	4
TPSA	113.56	133.99	75.43
Log K_P_ (skin permeation)	−8.11	−8.02	−6.44
Lipinski’s rule violation	No	No	No
Bioavailability score	0.55	0.55	0.55
GI absorption	High	High	High
PAINS alerts	0	0	0
P-pg substrate	Yes	No	Yes

## Data Availability

The authors confirm that the data supporting the study’s findings are included in the article and its Supplementary Information.

## Supplementary Materials

The following supporting information can be downloaded at https://www.mdpi.com/article/10.3390/ijms23095054/s1, References [54,55,56,57,58,59,60] are cited in the supplementary materials.
