# Supplementary material for: Toward the Discovery of a Novel Class of Leads for High Altitude Disorders by Virtual Screening and Molecular Dynamics Approaches Targeting Carbonic Anhydrase
Source: Int J Mol Sci. 2022 May 2;23(9):5054. doi: 10.3390/ijms23095054 (PMC9104310; doi:10.3390/ijms23095054)
Supplement: Supplementary file 1 [file ijms-23-05054-s001.zip › ijms-1683267-supplementary.pdf]

Supplementary Information

Toward the Discovery of a Novel Class of leads for high altitude disorders by Virtual Screening and molecular dynamics Approaches Targeting carbonic anhydrase

Amena Ali[1,2,*], Abuzer Ali[3], Musarrat Husain Warsi[4], Mohammad Akhlaquer Rahman[4], Mohamed Jawed Ahsan[5] and Faizul Azam[6]

[1] *High Altitude Research Center, Taif University, P.O. Box 11099, Taif 21944, Saudi Arabia; amrathore@tu.edu.sa*

[2] *Department of Pharmaceutical Chemistry, College of Pharmacy, Taif University, P.O. Box 11099, Taif 21944, Saudi Arabia; amrathore@tu.edu.sa*

[3] *Department of Pharmacognosy, College of Pharmacy, Taif University, P.O. Box 11099, Taif 21944, Saudi Arabia; abuali@tu.edu.sa*

[4] *Department of Pharmaceutics and Industrial Pharmacy, College of Pharmacy, Taif University, P.O. Box 11099, Taif 21944, Saudi Arabia; mvarsi@tu.edu.sa* (MHW); *mrahman@tu.edu.sa* (MAR)

[5] *Department of Pharmaceutical Chemistry, Maharishi Arvind College of Pharmacy, Jaipur, Rajasthan 302 039, India; jawedpharma@gmail.com*

[6] *Department of Pharmaceutical Chemistry & Pharmacognosy, Unaizah College of Pharmacy, Qassim University, Uniazah 51911, Saudi Arabia; f.azam@qu.edu.sa*

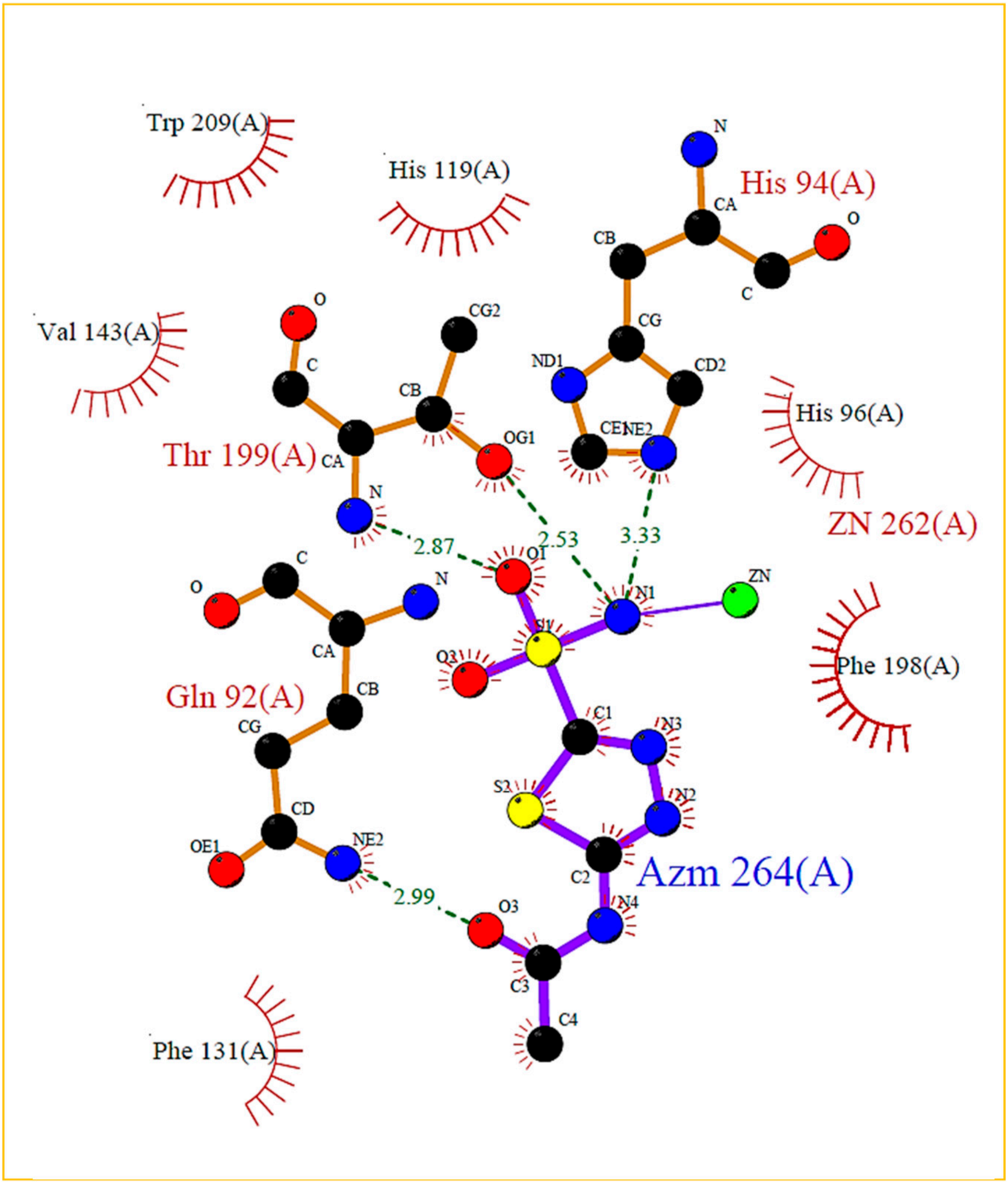

Figure S1. Ligplot diagram of co-crystallized ligand acetazolamide (PDB: 1YDB).

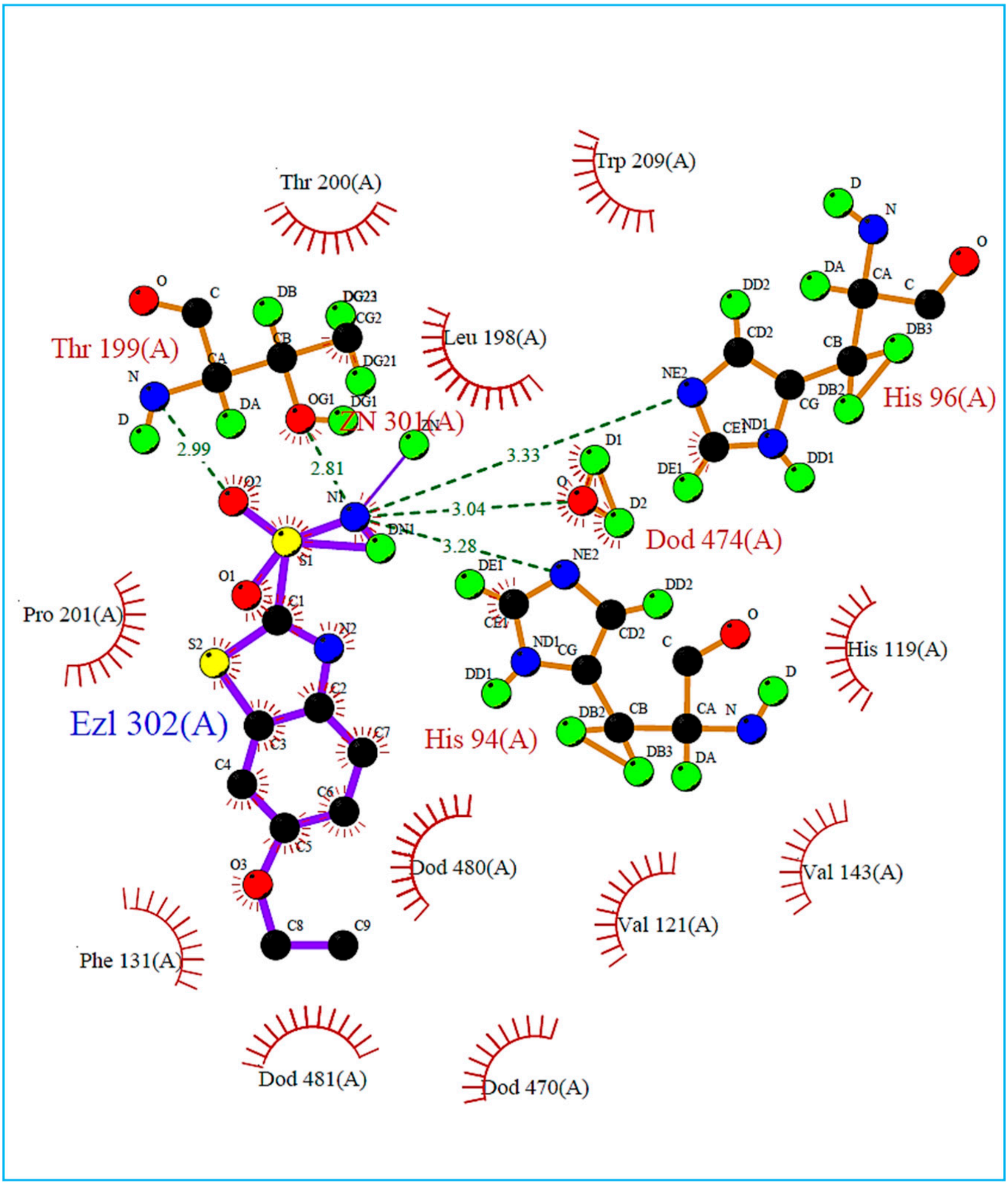

Figure S2. Ligplot diagram of co-crystallized ligand ethoxzolamide (PDB: 6BCC).

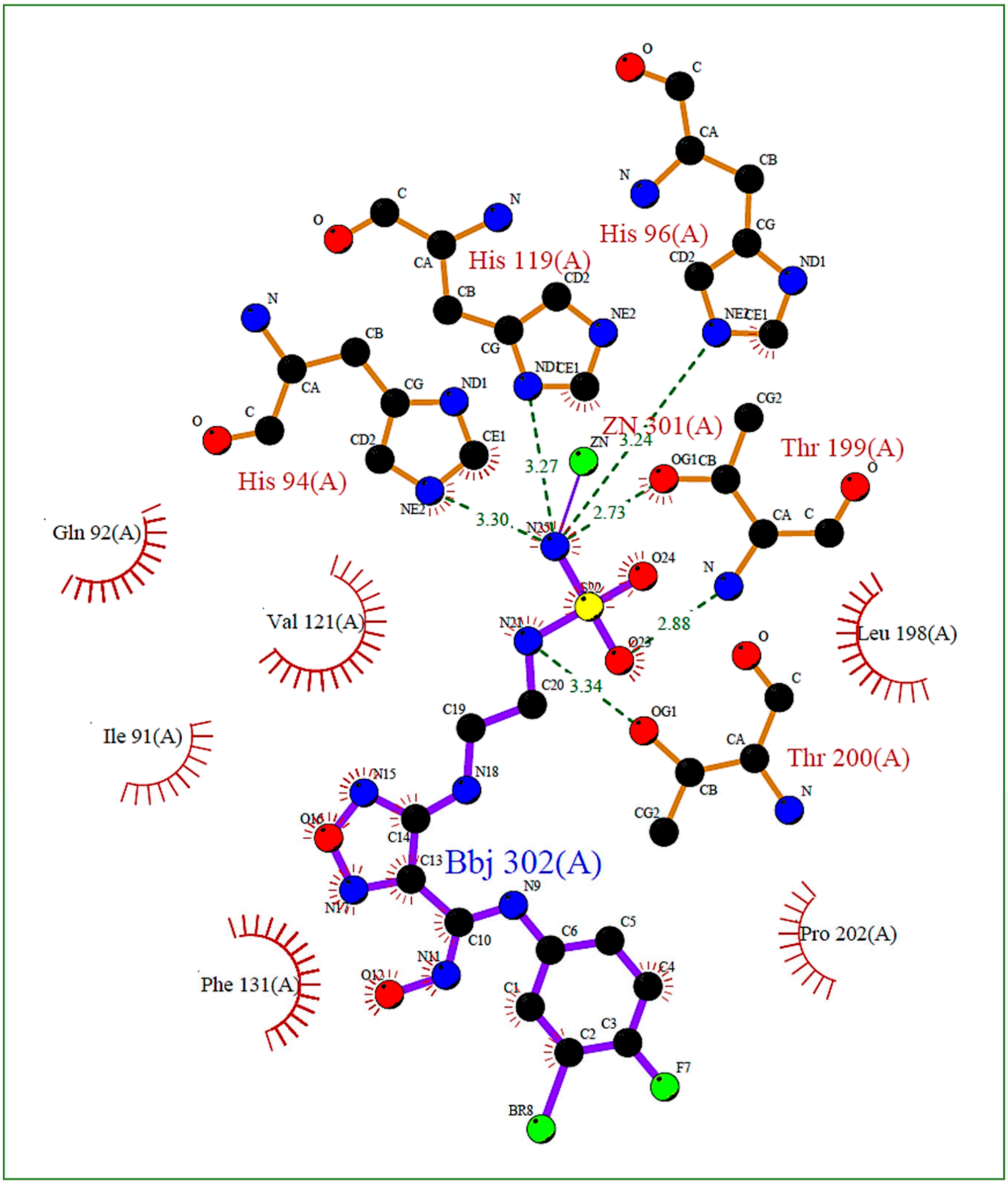

Figure S3. Ligplot diagram of co-crystallized ligand epacadostat (PDB: 6IC2).

Table S1. Carbonic anhydrase inhibitory activity of acetazolamide against different isoforms.

SN	Target	Ki (nM)	References
1	Carbonic anhydrase-1	800	Duffel et al, 1986 [1]
2	Carbonic anhydrase-2	0.012	Carta et al, 2016 [2]
3	Carbonic anhydrase-3	200000	Temperini et al, 2008 [3]
4	Carbonic anhydrase-4	12	Scozzafava et al, 2000 [4]
5	Carbonic anhydrase-7	2.5	Vullo et al, 2005 [5]
6	Carbonic anhydrase-12	5.6	Guzel-Akdemir et al, 2013 [6]
7	Carbonic anhydrase-14	41	Temperini et al, 2008 [7]

Table S2. Top-10 hits obtained from 1,3,4-thiadiazole-based pharmacophore.

Pharmacophore	Top-10 hits	Chemical structure	Docking score
	ZINC12336992	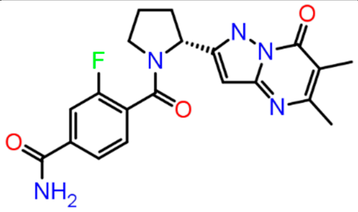	-9.0
	ZINC17094775	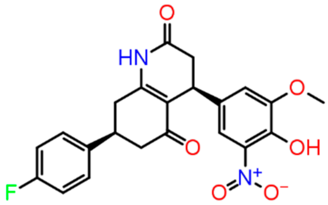	-8.8
	ZINC23767494	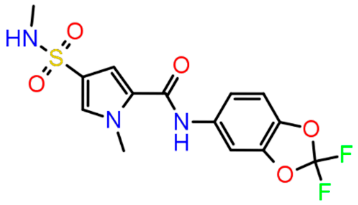	-8.3
	ZINC13095340	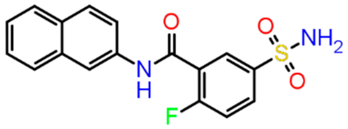	-8.3
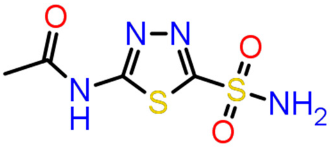	ZINC16598965	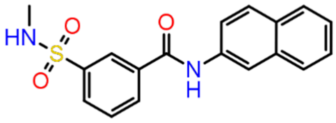	-8.2
	ZINC56363882	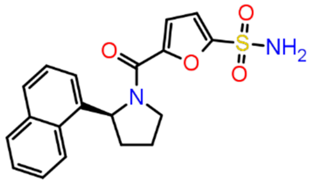	-8.1
	ZINC32892923	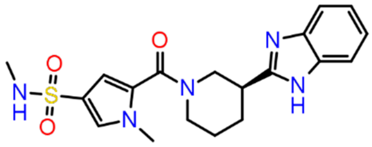	-7.9
	ZINC25132072	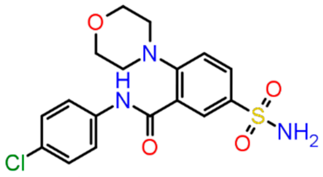	-7.9
	ZINC48332299	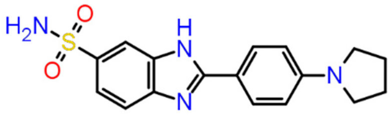	-7.9
	ZINC6977130	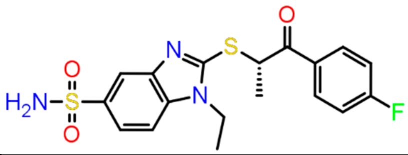	-7.9

Table S3. Top-10 hits obtained from 1,3-benzothiazole-based pharmacophore.

Pharmacophore	Top-10 hits	Chemical structure	Docking score
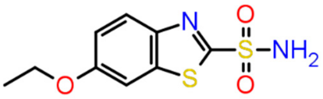	ZINC24751284	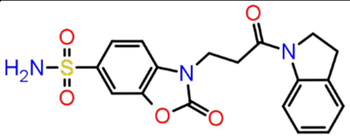	-9.0
	ZINC32809300	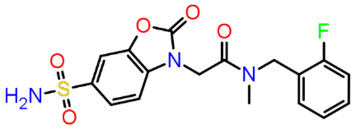	-8.6
	ZINC23318431	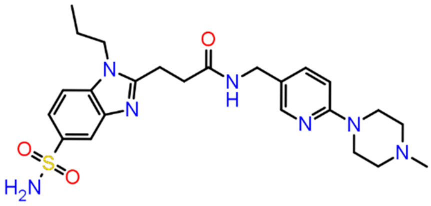	-8.5
	ZINC4090104	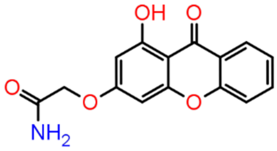	-8.4
	ZINC217474540	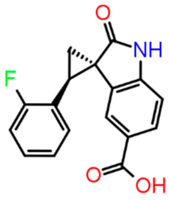	-8.3
	ZINC9724717	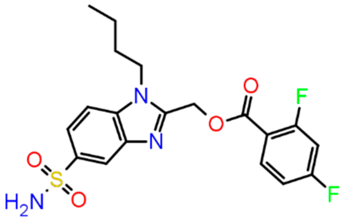	-8.3
	ZINC58168621	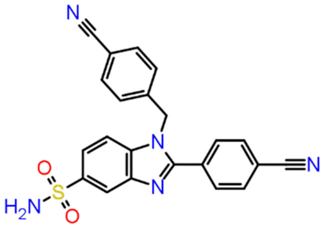	-8.3
	ZINC4088326	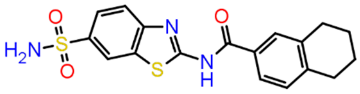	-8.2
	ZINC46034715	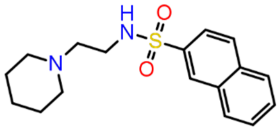	-8.1
	ZINC95443933	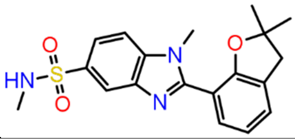	-8.1

Table S4. Top-10 hits screened from 1,2,5-oxadiazole-based pharmacophore.

Pharmacophore	Top-10 hits	Chemical structure	Docking score
	ZINC58324738	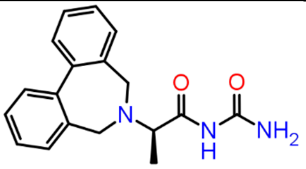	-8.9
	ZINC7016498	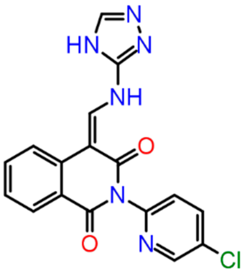	-8.3
	ZINC4704625	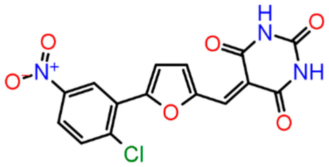	-7.7
	ZINC1506420260	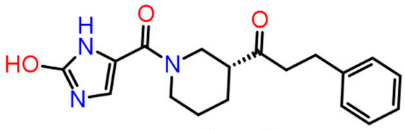	-7.6
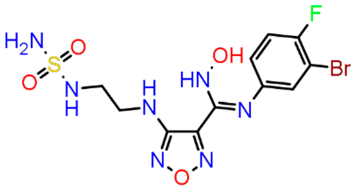	ZINC5669913	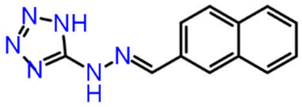	-7.6
	ZINC12819573	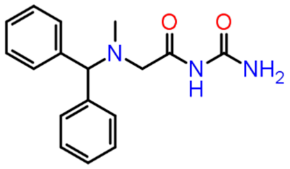	-7.5
	ZINC14093674	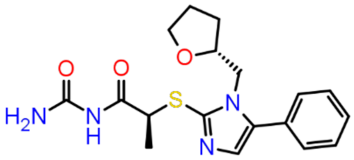	-7.5
	ZINC04624732	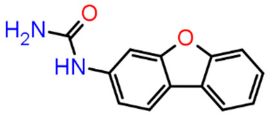	-7.5
	ZINC253405853	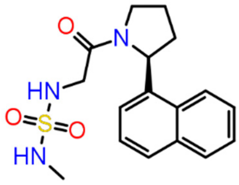	-7.1
	ZINC16399055	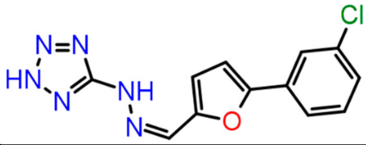	-7.0

Table S5. Cartesian coordinates (Å) of ZINC12336992 after DFT optimization.

Atom Number	Element	X	Y	Z
1	C	-3.241823	6.117653	11.580995
2	N	-3.706936	4.955625	12.363729
3	C	-3.859764	5.085262	13.716365
4	O	-3.497037	6.087488	14.312599
5	C	-4.115516	3.819206	11.508259
6	C	-3.549596	4.195466	10.133870
7	C	-3.629627	5.729345	10.133178
8	C	-1.764805	6.364687	11.752075
9	N	-0.912924	5.327159	12.031795
10	N	0.344838	5.843932	12.143054
11	C	0.336708	7.182560	11.834451
12	N	1.446948	7.893019	11.818236
13	C	2.582949	7.227919	12.157259
14	C	3.835876	8.067032	12.132184
15	C	2.651966	5.880841	12.517719
16	C	3.940575	5.199993	12.900041
17	C	1.462921	5.076989	12.535375
18	C	-1.033194	7.511558	11.580070
19	O	1.291198	3.895876	12.831541
20	C	-4.433987	3.917261	14.481673
21	C	-5.734730	3.449954	14.311185
22	F	-6.511944	4.013252	13.352909
23	C	-6.276339	2.443034	15.087772
24	C	-5.503022	1.875372	16.101678
25	C	-4.191991	2.325540	16.298279
26	C	-3.674625	3.344586	15.507315
27	C	-6.144161	0.777253	16.912955
28	N	-5.539901	0.476986	18.104795
29	H	-6.022279	-0.188447	18.689789
30	H	-4.933917	1.138473	18.561206
31	O	-7.130944	0.185194	16.514968
32	H	-3.761881	7.016096	11.917793
33	H	-3.714792	2.879922	11.894550
34	H	-5.204379	3.741307	11.473027
35	H	-2.510009	3.869706	10.043640
36	H	-4.116873	3.739621	9.320911
37	H	-4.656362	6.055538	9.943677
38	H	-2.980557	6.200289	9.394899
39	H	-1.100372	4.483124	12.556951
40	H	4.586770	7.643022	11.458014
41	H	4.294344	8.122558	13.125157
42	H	3.587678	9.074594	11.802210
43	H	3.731635	4.175032	13.206699
44	H	4.443966	5.711271	13.727147
45	H	4.647072	5.165439	12.062960
46	H	-1.400818	8.487618	11.312106
47	H	-7.288872	2.098214	14.922082
48	H	-3.558351	1.871711	17.050769
49	H	-2.669120	3.710871	15.677092

Table S6. Bond parameters of ZINC12336992 after DFT optimization.

No.	Definition	Bond length (in Å)	No.	Definition	Bond angles (in °)
1	B (N1, C0)	1.48	1	A (C6, C0, C7)	113.12
2	B (C2, N1)	1.37	2	A (C7, C0, H31)	107.28
3	B (O3, C2)	1.22	3	A (N1, C0, C6)	102.68
4	B (C4, N1)	1.48	4	A (N1, C0, C7)	112.18
5	B (C5, C4)	1.53	5	A (N1, C0, H31)	109.53
6	B (C6, C5)	1.54	6	A (C6, C0, H31)	112.07
7	B (C6, C0)	1.55	7	A (C2, N1, C4)	127.86
8	B (C7, C0)	1.51	8	A (C0, N1, C4)	112.64
9	B (N8, C7)	1.37	9	A (C0, N1, C2)	119.02
10	B (N9, N8)	1.36	10	A (N1, C2, O3)	121.84
11	B (C10, N9)	1.37	11	A (O3, C2, C19)	120.02
12	B (N11, C10)	1.32	12	A (N1, C2, C19)	118.07
13	B (C12, N11)	1.36	13	A (C5, C4, H33)	110.89
14	B (C13, C12)	1.51	14	A (H32, C4, H33)	108.42
15	B (C14, C12)	1.40	15	A (N1, C4, H33)	110.41
16	B (C15, C14)	1.51	16	A (N1, C4, C5)	103.17
17	B (C16, C14)	1.44	17	A (C5, C4, H32)	113.13
18	B (C16, N9)	1.41	18	A (N1, C4, H32)	110.78
19	B (C17, C10)	1.43	19	A (H34, C5, H35)	107.96
20	B (C17, C7)	1.37	20	A (C4, C5, C6)	103.07
21	B (O18, C16)	1.23	21	A (C6, C5, H35)	112.94
22	B (C19, C2)	1.51	22	A (C4, C5, H34)	110.60
23	B (C20, C19)	1.39	23	A (C6, C5, H34)	110.31
24	B (F21, C20)	1.36	24	A (C4, C5, H35)	111.93
25	B (C22, C20)	1.38	25	A (H36, C6, H37)	108.23
26	B (C23, C22)	1.40	26	A (C5, C6, H37)	113.62
27	B (C24, C23)	1.40	27	A (C0, C6, H37)	112.08
28	B (C25, C24)	1.39	28	A (C0, C6, C5)	103.71
29	B (C25, C19)	1.40	29	A (C5, C6, H36)	110.29
30	B (C26, C23)	1.51	30	A (C0, C6, H36)	108.80
31	B (N27, C26)	1.37	31	A (N8, C7, C17)	109.08
32	B (H28, N27)	1.01	32	A (C0, C7, C17)	130.22
33	B (H29, N27)	1.01	33	A (C0, C7, N8)	120.52
34	B (O30, C26)	1.22	34	A (C7, N8, N9)	107.62
35	B (H31, C0)	1.09	35	A (N9, N8, H38)	116.39
36	B (H32, C4)	1.09	36	A (C7, N8, H38)	128.47
37	B (H33, C4)	1.09	37	A (N8, N9, C10)	110.20
38	B (H34, C5)	1.09	38	A (C10, N9, C16)	126.63
39	B (H35, C5)	1.09	39	A (N8, N9, C16)	123.17
40	B (H36, C6)	1.09	40	A (N9, C10, N11)	121.53
41	B (H37, C6)	1.09	41	A (N11, C10, C17)	132.83
42	B (H38, N8)	1.01	42	A (N9, C10, C17)	105.63
43	B (H39, C13)	1.09	43	A (C10, N11, C12)	115.92
44	B (H40, C13)	1.10	44	A (C13, C12, C14)	120.00
45	B (H41, C13)	1.09	45	A (N11, C12, C14)	125.29
46	B (H42, C15)	1.09	46	A (N11, C12, C13)	114.70

47	B (H43, C15)	1.09	47	A (H40, C13, H41)	108.86
48	B (H44, C15)	1.10	48	A (H39, C13, H41)	109.16
49	B (H45, C17)	1.08	49	A (C12, C13, H41)	109.30
50	B (H46, C22)	1.08	50	A (C12, C13, H39)	111.38
51	B (H47, C24)	1.08	51	A (H39, C13, H40)	106.91
52	B (H48, C25)	1.08	52	A (C12, C13, H40)	111.16
			53	A (C12, C14, C15)	122.94
			54	A (C12, C14, C16)	120.17
			55	A (C15, C14, C16)	116.89
			56	A (H43, C15, H44)	107.18
			57	A (H42, C15, H44)	107.99
			58	A (H42, C15, H43)	108.34
			59	A (C14, C15, H43)	111.95
			60	A (C14, C15, H42)	109.41
			61	A (C14, C15, H44)	111.82
			62	A (C14, C16, O18)	131.04
			63	A (N9, C16, O18)	118.57
			64	A (N9, C16, C14)	110.39
			65	A (C7, C17, C10)	107.22
			66	A (C10, C17, H45)	125.39
			67	A (C7, C17, H45)	127.39
			68	A (C20, C19, C25)	117.35
			69	A (C2, C9, C25)	118.81
			70	A (C2, C9, C20)	123.56
			71	A (F21, C20, C22)	118.36
			72	A (C19, C20, C22)	122.80
			73	A (C19, C20, F21)	118.84
			74	A (C23, C22, H46)	119.99
			75	A (C20, C22, H46)	120.84
			76	A (C20, C22, C23)	119.16
			77	A (C22, C23, C26)	116.83
			78	A (C24, C23, C26)	123.82
			79	A (C22, C23, C24)	119.34
			80	A (C23, C24, H47)	120.70
			81	A (C25, C24, H47)	118.99
			82	A (C23, C24, C25)	120.29
			83	A (C24, C25, H48)	120.27
			84	A (C19, C25, H48)	118.70
			85	A (C19, C25, C24)	121.02
			86	A (N27, C26, O30)	122.37
			87	A (C23, C26, O30)	121.51
			88	A (C23, C26, N27)	116.11
			89	A (H28, N27, H29)	117.29
			90	A (C26, N27, H29)	121.07
			91	A (C26, N27, H28)	115.99

Table S7. Cartesian coordinates (Å) of ZINC24751284 after DFT optimization.

Atom Number	Element	X	Y	Z
1	C	-5.072096	2.042018	16.310934
2	C	-3.825848	2.677710	16.233932
3	C	-3.825489	3.918686	15.642094
4	C	-4.986218	4.522452	15.143721
5	N	-4.585585	5.751019	14.638263
6	C	-3.218720	5.900902	14.811162
7	O	-2.521030	6.819968	14.489912
8	C	-6.217954	3.888519	15.237290
9	O	-2.753472	4.753139	15.444687
10	C	-6.244838	2.625270	15.832909
11	S	-5.149148	0.405023	17.038179
12	O	-6.505422	0.203061	17.538563
13	O	-3.964062	0.241834	17.874756
14	N	-4.960403	-0.622709	15.707652
15	H	-4.105902	-1.161583	15.799598
16	H	-5.774486	-1.219916	15.610018
17	C	-5.411276	6.766833	13.989016
18	C	-5.133752	6.857484	12.485987
19	C	-6.188731	7.718611	11.793486
20	N	-5.916971	8.078639	10.498703
21	C	-6.788644	8.804120	9.643889
22	C	-6.173608	9.011813	8.404290
23	C	-6.839236	9.693633	7.397281
24	C	-8.131454	10.169156	7.635377
25	C	-8.732882	9.961463	8.875315
26	C	-4.774746	8.442721	8.414752
27	C	-8.074696	9.278482	9.900850
28	C	-4.719451	7.643394	9.736383
29	O	-7.215804	8.040926	12.374465
30	H	-2.929338	2.226276	16.636488
31	H	-7.127702	4.347618	14.872316
32	H	-7.183417	2.098702	15.947243
33	H	-6.457244	6.525679	14.167157
34	H	-5.214552	7.728239	14.466549
35	H	-4.127155	7.248900	12.326532
36	H	-5.155118	5.853847	12.042804
37	H	-6.362031	9.856619	6.436470
38	H	-8.664546	10.700891	6.855591
39	H	-9.735079	10.334689	9.054990
40	H	-4.569984	7.804069	7.552522
41	H	-4.028964	9.244496	8.409597
42	H	-8.534893	9.117429	10.862844
43	H	-3.809040	7.846247	10.300009
44	H	-4.772012	6.565632	9.550424

Table S8. Bond parameters of ZINC24751284 after DFT optimization.

No.	Definition	Bond length (in Å)	No.	Definition	Bond angles (in °)
1	B (C1, C0)	1.40	1	A (C1, C0, S10)	118.34
2	B (C2, C1)	1.37	2	A (C1, C0, C9)	122.65
3	B (C3, C2)	1.40	3	A (C9, C0, S10)	119.00
4	B (N4, C3)	1.39	4	A (C0, C1, H29)	121.84
5	B (C5, N4)	1.39	5	A (C2, C1, H29)	122.47
6	B (O6, C5)	1.20	6	A (C0, C1, C2)	115.68
7	B (C7, C3)	1.39	7	A (C3, C2, O8)	109.52
8	B (O8, C5)	1.39	8	A (C1, C2, O8)	127.64
9	B (O8, C2)	1.37	9	A (C1, C2, C3)	122.84
10	B (C9, C7)	1.40	10	A (C2, C3, N4)	105.79
11	B (C9, C0)	1.39	11	A (N4, C3, C7)	133.23
12	B (S10, C0)	1.79	12	A (C2, C3, C7)	120.97
13	B (O11, S10)	1.46	13	A (C5, N4, C16)	122.52
14	B (O12, S10)	1.46	14	A (C3, N4, C5)	109.58
15	B (N13, S10)	1.69	15	A (C3, N4, C16)	127.90
16	B (H14, N13)	1.01	16	A (N4, C5, O8)	107.30
17	B (H15, N13)	1.01	17	A (N4, C5, O6)	128.61
18	B (C16, N4)	1.46	18	A (O6, C5, O8)	124.09
19	B (C17, C16)	1.53	19	A (C3, C7, H30)	121.95
20	B (C18, C17)	1.53	20	A (C9, C7, H30)	120.74
21	B (N19, C18)	1.37	21	A (C3, C7, C9)	117.30
22	B (C20, N19)	1.42	22	A (C2, O8, C5)	107.81
23	B (C21, C20)	1.40	23	A (C7, C9, H31)	120.11
24	B (C22, C21)	1.39	24	A (C0, C9, H31)	119.32
25	B (C23, C22)	1.40	25	A (C0, C9, C7)	120.55
26	B (C24, C23)	1.39	26	A (O12, S10, N13)	106.99
27	B (C25, C21)	1.51	27	A (O11, S10, N13)	106.81
28	B (C26, C24)	1.40	28	A (O11, S10, O12)	122.85
29	B (C26, C20)	1.39	29	A (C0, S10, N13)	103.35
30	B (C27, C25)	1.55	30	A (C0, S10, O12)	107.44
31	B (C27, N19)	1.48	31	A (C0, S10, O11)	107.78
32	B (O28, C18)	1.22	32	A (H14, N13, H15)	111.84
33	B (H29, C1)	1.08	33	A (S10, N13, H15)	110.11
34	B (H30, C7)	1.08	34	A (S10, N13, H14)	110.21
35	B (H31, C9)	1.08	35	A (N4, C16, C17)	112.02
36	B (H32, C16)	1.09	36	A (N4, C16, H33)	108.43
37	B (H33, C16)	1.09	37	A (C17, C16, H32)	110.37
38	B (H34, C17)	1.09	38	A (N4, C16, H32)	108.44
39	B (H35, C17)	1.10	39	A (H32, C16, H33)	107.27
40	B (H36, C22)	1.09	40	A (C17, C16, H33)	110.16
41	B (H37, C23)	1.08	41	A (C16, C17, H35)	109.80
42	B (H38, C24)	1.08	42	A (C16, C17, H34)	109.37
43	B (H39, C25)	1.09	43	A (C16, C17, C18)	110.68
44	B (H40, C25)	1.10	44	A (C18, C17, H35)	108.60
45	B (H41, C26)	1.08	45	A (H34, C17, H35)	106.67
46	B (H42, C27)	1.09	46	A (C18, C17, H34)	111.62

47	B (H43, C27)	1.09	47	A (C17, C18, N19)	116.05
			48	A (N19, C18, O28)	123.08
			49	A (C17, C18, O28)	120.87
			50	A (C20, N19, C27)	109.62
			51	A (C18, N19, C27)	124.59
			52	A (C18, N19, C20)	125.51
			53	A (C21, C20, C26)	121.20
			54	A (N19, C20, C21)	109.83
			55	A (N19, C20, C26)	128.96
			56	A (C22, C21, C25)	129.42
			57	A (C20, C21, C25)	110.19
			58	A (C20, C21, C22)	120.36
			59	A (C21, C22, C23)	119.18
			60	A (C23, C22, H36)	120.43
			61	A (C21, C22, H36)	120.39
			62	A (C22, C23, C24)	120.00
			63	A (C24, C23, H37)	120.06
			64	A (C22, C23, H37)	119.94
			65	A (C23, C24, C26)	121.51
			66	A (C26, C24, H38)	118.82
			67	A (C23, C24, H38)	119.67
			68	A (C21, C25, C27)	103.53
			69	A (C27, C25, H40)	111.00
			70	A (C21, C25, H40)	110.79
			71	A (C27, C25, H39)	111.46
			72	A (C21, C25, H39)	112.86
			73	A (H39, C25, H40)	107.26
			74	A (C20, C26, C24)	117.74
			75	A (C24, C26, H41)	121.79
			76	A (C20, C26, H41)	120.47
			77	A (N19, C27, C25)	104.99
			78	A (H42, C27, H43)	108.13
			79	A (C25, C27, H43)	111.23
			80	A (N19, C27, H43)	109.69
			81	A (C25, C27, H42)	112.08
			82	A (N19, C27, H42)	110.71

Table S9. Cartesian coordinates (Å) of ZINC58324738 after DFT optimization.

Atom Number	Element	X	Y	Z
1	N	-2.685425	4.320410	12.858553
2	C	-3.733304	3.274254	12.891606
3	C	-4.831087	3.452227	13.924990
4	C	-5.788692	4.476022	13.754100
5	C	-6.828423	4.595690	14.686230
6	C	-6.928399	3.724267	15.766302
7	C	-5.985201	2.712679	15.931564
8	C	-5.695827	5.400697	12.593343
9	C	-4.451627	5.950243	12.216539
10	C	-4.382139	6.791895	11.104810
11	C	-5.522120	7.107764	10.369458
12	C	-6.753249	6.576524	10.746159
13	C	-6.835446	5.728714	11.846553
14	C	-4.946463	2.584053	15.012709
15	C	-3.222013	5.680873	13.055166
16	C	-1.465467	4.043982	13.641845
17	C	-1.473516	4.405235	15.134527
18	C	-0.209501	4.655657	12.952787
19	N	-0.393288	4.809432	11.586262
20	H	-1.347968	4.598825	11.305063
21	C	0.514404	5.339051	10.649990
22	O	1.589489	5.814546	10.914816
23	N	-0.007204	5.299509	9.363099
24	H	0.671452	5.522423	8.650641
25	H	-0.658505	4.568430	9.119196
26	O	0.800948	4.896509	13.565209
27	H	-4.200274	3.246914	11.901366
28	H	-3.239784	2.311337	13.034879
29	H	-7.554034	5.393388	14.570564
30	H	-7.737371	3.838470	16.479260
31	H	-6.057935	2.027698	16.768971
32	H	-3.423300	7.213650	10.820078
33	H	-5.449298	7.766615	9.511399
34	H	-7.647021	6.813709	10.179727
35	H	-7.790649	5.294475	12.120623
36	H	-4.219460	1.786759	15.134417
37	H	-3.479931	5.857210	14.108844
38	H	-2.437598	6.396042	12.793185
39	H	-1.303116	2.962067	13.561139
40	H	-0.533335	4.085403	15.583715
41	H	-2.301490	3.912952	15.648559
42	H	-1.553833	5.482399	15.292755

Table S10. Bond parameters of ZINC58324738 after DFT optimization.

No.	Definition	Bond length (in Å)	No.	Definition	Bond angles (in °)
1	B (C1, N0)	1.48	1	A (C1, N0, C15)	116.15
2	B (C2, C1)	1.52	2	A (C1, N0, C14)	113.02
3	B (C3, C2)	1.41	3	A (C14, N0, C15)	113.74
4	B (C4, C3)	1.40	4	A (C2, C1, H26)	108.06
5	B (C5, C4)	1.39	5	A (N0, C1, C2)	116.36
6	B (C6, C5)	1.39	6	A (H26, C1, H27)	106.83
7	B (C7, C3)	1.49	7	A (C2, C1, H27)	109.94
8	B (C8, C7)	1.41	8	A (N0, C1, H27)	107.83
9	B (C9, C8)	1.40	9	A (N0, C1, H26)	107.41
10	B (C10, C9)	1.39	10	A (C1, C2, C3)	119.54
11	B (C11, C10)	1.39	11	A (C3, C2, C13)	119.27
12	B (C12, C11)	1.39	12	A (C1, C2, C13)	121.14
13	B (C12, C7)	1.40	13	A (C2, C3, C7)	120.19
14	B (C13, C6)	1.39	14	A (C4, C3, C7)	120.83
15	B (C13, C2)	1.40	15	A (C2, C3, C4)	118.98
16	B (C14, C8)	1.51	16	A (C5, C4, H28)	119.69
17	B (C14, N0)	1.48	17	A (C3, C4, H28)	119.22
18	B (C15, N0)	1.48	18	A (C3, C4, C5)	121.07
19	B (C16, C15)	1.54	19	A (C6, C5, H29)	120.24
20	B (C17, C15)	1.56	20	A (C4, C5, H29)	119.87
21	B (N18, C17)	1.39	21	A (C4, C5, C6)	119.89
22	B (H19, N18)	1.02	22	A (C5, C6, H30)	120.33
23	B (C20, N18)	1.41	23	A (C13, C6, H30)	120.08
24	B (O21, C20)	1.21	24	A (C5, C6, C13)	119.59
25	B (N22, C20)	1.39	25	A (C3, C7, C12)	120.71
26	B (H23, N22)	1.01	26	A (C8, C7, C12)	118.91
27	B (H24, N22)	1.01	27	A (C3, C7, C8)	120.37
28	B (O25, C17)	1.21	28	A (C9, C8, C14)	120.56
29	B (H26, C1)	1.10	29	A (C7, C8, C14)	119.95
30	B (H27, C1)	1.09	30	A (C7, C8, C9)	119.42
31	B (H28, C4)	1.08	31	A (C10, C9, H31)	119.76
32	B (H29, C5)	1.08	32	A (C8, C9, H31)	119.15
33	B (H30, C6)	1.08	33	A (C8, C9, C10)	121.09
34	B (H31, C9)	1.09	34	A (C11, C10, H32)	120.34
35	B (H32, C10)	1.08	35	A (C9, C10, H32)	120.04
36	B (H33, C11)	1.08	36	A (C9, C10, C11)	119.61
37	B (H34, C12)	1.08	37	A (C10, C11, H33)	120.25
38	B (H35, C13)	1.09	38	A (C10, C11, C12)	119.90
39	B (H36, C14)	1.10	39	A (C12, C11, H33)	119.84
40	B (H37, C14)	1.09	40	A (C7, C12, H34)	119.20
41	B (H38, C15)	1.10	41	A (C7, C12, C11)	121.06
42	B (H39, C16)	1.09	42	A (C11, C12, H34)	119.72
43	B (H40, C16)	1.09	43	A (C2, C13, H35)	119.24
44	B (H41, C16)	1.09	44	A (C2, C13, C6)	121.20
			45	A (C6, C13, H35)	119.55
			46	A (C8, C14, H36)	108.19

47	A (N0, C14, H36)	111.16
48	A (C8, C14, H37)	109.51
49	A (N0, C14, C8)	112.70
50	A (H36, C14, H37)	107.05
51	A (N0, C14, H37)	108.07
52	A (C16, C15, C17)	109.99
53	A (C17, C15, H38)	103.62
54	A (N0, C15, C17)	110.99
55	A (N0, C15, C16)	117.87
56	A (C16, C15, H38)	107.71
57	A (N0, C15, H38)	105.55
58	A (H39, C16, H41)	107.05
59	A (C15, C16, H41)	111.92
60	A (H39, C16, H40)	109.14
61	A (C15, C16, H40)	110.82
62	A (C15, C16, H39)	109.09
63	A (H40, C16, H41)	108.71
64	A (C15, C17, N18)	111.87
65	A (N18, C17, O25)	126.09
66	A (C15, C17, O25)	121.97
67	A (C17, N18, C20)	127.69
68	A (C17, N18, H19)	111.94
69	A (H19, N18, C20)	119.95
70	A (O21, C20, N22)	123.36
71	A (N18, C20, N22)	111.31
72	A (N18, C20, O21)	125.29
73	A (H23, N22, H24)	115.06
74	A (C20, N22, H24)	119.14
75	A (C20, N22, H23)	113.29

References

1. Duffel, M.W.; Ing, I.S.; Segarra, T.M.; Dixson, J.A.; Barfknecht, C.F.; Schoenwald, R.D. N-Substituted sulfonamide carbonic anhydrase inhibitors with topical effects on intraocular pressure. *J. Med. Chem.* **1986**, *29*, 1488–1494, doi:10.1021/jm00158a028.

2. Carta, F.; Ferraroni, M.; Scozzafava, A.; Supuran, C.T. Fluorescent sulfonamide carbonic anhydrase inhibitors incorporating 1,2,3-triazole moieties: Kinetic and X-ray crystallographic studies. *Bioorg. Med. Chem.* **2016**, *24*, 104–112, doi:https://doi.org/10.1016/j.bmc.2015.11.031.

3. Temperini, C.; Cecchi, A.; Scozzafava, A.; Supuran, C.T. Carbonic anhydrase inhibitors. Interaction of indapamide and related diuretics with 12 mammalian isozymes and X-ray crystallographic studies for the indapamide–isozyme II adduct. *Bioorg. Med. Chem. Lett.* **2008**, *18*, 2567–2573, doi:https://doi.org/10.1016/j.bmcl.2008.03.051.

4. Scozzafava, A.; Menabuoni, L.; Mincione, F.; Briganti, F.; Mincione, G.; Supuran, C.T. Carbonic Anhydrase Inhibitors: Perfluoroalkyl/Aryl-Substituted Derivatives of Aromatic/Heterocyclic Sulfonamides as Topical Intraocular Pressure-Lowering Agents

with Prolonged Duration of Action. *J. Med. Chem.* **2000**, *43*, 4542–4551, doi:10.1021/jm000296j.

5. Vullo, D.; Voipio, J.; Innocenti, A.; Rivera, C.; Ranki, H.; Scozzafava, A.; Kaila, K.; Supuran, C.T. Carbonic anhydrase inhibitors. Inhibition of the human cytosolic isozyme VII with aromatic and heterocyclic sulfonamides. *Bioorg. Med. Chem. Lett.* **2005**, *15*, 971–976, doi:https://doi.org/10.1016/j.bmcl.2004.12.052.

6. Güzel-Akdemir, Ö.; Akdemir, A.; Isik, S.; Vullo, D.; Supuran, C.T. o-Benzenedisulfonimido–sulfonamides are potent inhibitors of the tumor-associated carbonic anhydrase isoforms CA IX and CA XII. *Bioorg. Med. Chem.* **2013**, *21*, 1386–1391, doi:https://doi.org/10.1016/j.bmc.2012.12.037.

7. Temperini, C.; Innocenti, A.; Scozzafava, A.; Supuran, C.T. Carbonic anhydrase inhibitors. Interaction of the antitumor sulfamate EMD 486019 with twelve mammalian carbonic anhydrase isoforms: Kinetic and X-ray crystallographic studies. *Bioorg. Med. Chem. Lett.* **2008**, *18*, 4282–4286, doi:https://doi.org/10.1016/j.bmcl.2008.06.105.
